# Epigenetic Regulation of Nucleotide Excision Repair

**DOI:** 10.3389/fcell.2022.847051

**Published:** 2022-04-08

**Authors:** Wentao Li, Kyle Jones, Tyler J. Burke, Md Akram Hossain, Leah Lariscy

**Affiliations:** Department of Environmental Health Science, College of Public Health, University of Georgia, Athens, GA, United States

**Keywords:** epigenetics, nucleotide excision repair, DNA damage, histone modifications, genome architecture, chromatin remodeler

## Abstract

Genomic DNA is constantly attacked by a plethora of DNA damaging agents both from endogenous and exogenous sources. Nucleotide excision repair (NER) is the most versatile repair pathway that recognizes and removes a wide range of bulky and/or helix-distorting DNA lesions. Even though the molecular mechanism of NER is well studied through *in vitro* system, the NER process inside the cell is more complicated because the genomic DNA in eukaryotes is tightly packaged into chromosomes and compacted into a nucleus. Epigenetic modifications regulate gene activity and expression without changing the DNA sequence. The dynamics of epigenetic regulation play a crucial role during the *in vivo* NER process. In this review, we summarize recent advances in our understanding of the epigenetic regulation of NER.

## Introduction

The genome is essential for the survival of all living organisms and its integrity is critical for accurate transmission of genetic information to offspring. However, genomic DNA is constantly attacked by a plethora of DNA damaging agents both from endogenous and exogenous sources; For example, the reactive oxygen species (e.g., superoxide) produced in cellular metabolic processes, environmental carcinogens such as ultraviolet (UV) light, polycyclic aromatic hydrocarbons (PAHs) and mycotoxins, and high-frequency ionizing radiation like X-rays and gamma rays can directly distort the structure of DNA double helix and/or break the DNA strand(s) (Hoeijmakers, 2001). DNA lesions can block genome transcription and replication, which threatens the viability of damaged cells, or the whole organism, and eventually leads to mutations or chromosomal aberrations if not repaired in a timely and efficient manner. To deal with DNA lesions, organisms have evolved a complex system including DNA damage response and a variety of DNA repair pathways. There are five major DNA repair mechanisms: direct reversal repair, base excision repair, nucleotide excision repair (NER), mismatch repair, and double-strand break repair.

Among the five major repair mechanisms, NER is the most versatile one as it recognizes and removes a wide range of bulky and/or helix-distorting DNA lesions such as UV-induced cyclobutane pyrimidine dimers (CPDs) and (6-4) pyrimidine-pyrimidone photoproducts [(6-4)PPs] (Sancar, 2016). The basic NER process involves DNA lesion recognition, dual incision bracketing the lesion, release of excision product, repair synthesis, and ligation (Hu et al., 2017). NER consists of two subpathways: global genomic repair and transcription-coupled repair (TCR). Global genomic repair removes DNA lesions throughout the whole genome, while TCR acts only on the transcribed strand of actively transcribed genes (Mellon et al., 1987; Hanawalt and Spivak, 2008). They differ at the step of DNA lesion recognition but share the same repair machinery for the following repair process. The biochemical basics of global genomic repair were reconstituted *in vitro* for both prokaryotes and eukaryotes (Sancar, 2016). Six repair proteins, UvrA, UvrB, UvrC, UvrD, DNA Pol I, and DNA ligase, are required for global genomic repair in *E. coli* (Sancar and Rupp, 1983; Husain et al., 1985; Sancar and Tang, 1993). For global genomic repair in humans, six core repair factors, RPA, XPA, XPC, TFIIH, XPG, and XPF-ERCC1, are essential for damage recognition, dual incision, and release of the excision product in an *in vitro* system (Aboussekhra et al., 1995; Mu et al., 1995). Then, DNA Pol δ/ε and DNA ligase I or XRCC1-ligase III complex perform the repair synthesis and ligation respectively (Sancar, 1996; Wood, 1997). In contrast, even though TCR mechanism in *E. coli* has been elucidated *in vitro* (Selby and Sancar, 1993), TCR reaction in eukaryotes has not been reconstituted with purified protein components because of its complexity. The stalling of elongating RNA polymerase II (RNAP II) at a DNA lesion triggers TCR (Li et al., 2014b), and cockayne syndrome group B (CSB), the human homolog of yeast Rad26, binds to the lesion-stalled RNAP II and sequentially recruits CSA, UVSSA, and TFIIH to initiate NER in a cooperative manner (van der Weegen et al., 2020). Besides the above TCR factors, a variety of factors such as Sen1 (Li et al., 2016), Spt4/5 (Li et al., 2014a) and PFAc (Tatum et al., 2011), which directly interact with RNAP II, have been discovered to either facilitate or repress TCR in yeast (Li and Li, 2017). Recently, another elongation factor, ELOF1 (Prather et al., 2005), was found to facilitate TCR through promoting UVSSA and TFIIH recruitment (Olivieri et al., 2020; Geijer et al., 2021; van der Weegen et al., 2021). Genetic defects in NER genes are associated with a broad range of human diseases including xeroderma pigmentosum (XP), cockayne syndrome (CS), UV-sensitive syndrome (UV^S^S) (Cleaver and Thomas, 1993), and trichothiodystrophy (TTD).

**FIGURE 1:**
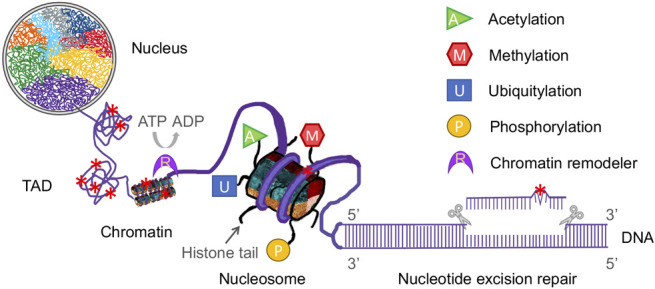
Diagrammatic representation of the complex components of epigenetics and nucleotide excision repair in a eukaryotic nucleus. Eukaryotic genomic DNA is wrapped around a histone octamer to form a nucleosome, which is the repeating unit of the chromatin. Chromatin fiber is folded into a hierarchy of loops and coils to form topologically associating domains (TADs). In this way, eukaryotic DNA is compacted into chromosomes in a nucleus. Each chromosome has its own territory (shown as different colors) in the nucleus. Nucleotide excision repair (NER) removes a wide range of DNA damage (denoted as red star) in cellular DNA and NER machinery requires access to damaged DNA in chromatin. Modifications of histone tails, such as acetylation, methylation, ubiquitylation and phosphorylation (shown as various colored shapes), and chromatin remodeling catalyzed by remodelers (shown as a purple crescent) affect the genome architecture and thus regulate the NER process.

Even though the mode of dual incision *in vivo* for NER is the same as *in vitro* studies (Kemp et al., 2012; Hu et al., 2013), the NER process in cells is far more complicated than that of *in vitro* experiments. The reason lies in the fact that genomic DNA in eukaryotes is tightly packaged into chromosomes and compacted into a nucleus, while *in vitro* system uses naked DNA template and purified repair proteins. It is much easier for repair proteins to access damage site in naked DNA than it is to access that in nucleosomal DNA (Schieferstein and Thoma, 1998; Hara et al., 2000; Liu and Smerdon, 2000). The human genome in a diploid cell contains around 6 billion DNA base pairs (bp) with a length of 3 m, and about 146 bp DNA is wrapped around a histone octamer (H3, H4, H2A, H2B) to form a nucleosome core particle (NCP), the fundamental repeating unit of the chromatin (Luger et al., 1997). NCPs are connected by linker DNA (10–70 bp) to form a 11 nm diameter “beads on a string” array. With the addition of linker histones (H1 and H5), which bind the nucleosome at the entry/exit sites of the linker DNA, the nucleosomal array is further consolidated into a 30 nm diameter chromatin fiber (Li and Reinberg, 2011). The arrangement of chromatin fiber in three-dimensional (3D) space within the nucleus is not random. Instead, chromatin fiber is folded into a hierarchy of loops and coils with the aid of scaffold proteins in different nuclear regions, forming specific territories such as topologically associating domains (TADs) and lamina-associated domains (LADs). In this way, the entire human genome is compacted into 23 pairs of chromosomes. Each chromosome occupies a unique part of the nuclear space termed chromosome territory (Meaburn and Misteli, 2007). The presence of nucleosomes, chromatin fiber, and higher-order spatial organization chromatin domains poses barriers to the NER repair proteins because the NER process requires the repair machinery to have access to DNA lesions to allow the sequential assembly and actions of repair complexes (Figure 1). Not only that, but epigenetic regulation, which modulates gene expression without changing the DNA sequence, can also affect the entire *in vivo* NER process (Li, 2012). In its broadest definition, which includes both heritable and non-heritable changes in gene activity and expression, epigenetic regulation consists of histone modifications, chromatin remodeling, nucleosome positioning, DNA modifications, and non-coding RNA. It is complex, dynamic, and important for transcription, DNA replication, and DNA repair. Meanwhile, DNA damage formation and repair can also affect epigenetic activities.

The dynamics of epigenetic regulation, which induces alterations of DNA lesion accessibility for repair machinery controlled by changes in spatial genome architecture, play a crucial role during the *in vivo* NER process (Dinant et al., 2008; Deem et al., 2012; Papamichos-Chronakis and Peterson, 2013; Karakaidos et al., 2020). In recent years, newly developed methodologies derived from next-generation sequencing (NGS) technology have been used for epigenomic profiling (Mehrmohamadi et al., 2021), genome-wide mapping of DNA damage and repair (Li and Sancar, 2020), and capturing spatial genome organization (Oluwadare et al., 2019). In addition, structural studies using cutting-edge cryo-electron microscopy (cryo-EM) and computer simulation modeling have revealed key insights into the NER process (Xu et al., 2017; Yan et al., 2021). In this review, we summarize recent advances in the epigenetic regulation of NER, focusing on insights into how the dynamics of genome architecture affect NER.

## Diverse Roles of Histone Modifications in NER

Through a hierarchy of compaction, the entire human genomic DNA is tightly packaged within a nucleus with a diameter of 5–10 μm (Misteli, 2007). In contrast to *in vitro* reconstitution assay system, the cellular NER machinery must overcome obstacles introduced by DNA packaging to gain access to DNA lesions before and during the occurrence of NER. Furthermore, the spatial genome architecture must be restored upon completion of repair (Smerdon, 1991; Polo et al., 2006; Tiwari et al., 2017). The HIRA (histone regulator A) complex, a replication-independent histone chaperone, has been shown to play important roles in chromatin restoration and transcription recovery after DNA damage and repair (Bouvier et al., 2021; Caron et al., 2021). The genome organization at different hierarchical levels can affect DNA damage formation as well. For example, at the single-nucleosome level, distribution of CPDs shows a striking 10.3-base periodicity (Gale et al., 1987) and (6-4)PPs are enriched in nucleosome linker regions (Mitchell et al., 1990). Recent studies have revealed that CPDs formation is significantly higher at “out” rotational settings in a nucleosome (Mao et al., 2016; Mao et al., 2017). Histone modifications, the covalent post-translational modifications (PTMs) of both histone tails and the core of the histone octamer, have been shown to play diverse roles in NER by altering chromatin structure (Li, 2012; Mao and Wyrick, 2016). These modifications include acetylation, methylation, phosphorylation, ubiquitylation, sumoylation, ADP-ribosylation, neddylation, and citrullination. Distinct histone modifications act alone or in combination to form the so-called “histone code” (Jenuwein and Allis, 2001). They regulate most, if not all, chromatin-templated cellular processes, such as gene transcription, DNA replication, DNA repair, and chromosome condensation, by disrupting chromatin contacts and/or recruiting nonhistone factors to chromatin (Kouzarides, 2007). There are additional histone modifications that are still being discovered. Here, we focus on the four major modifications (acetylation, methylation, ubiquitylation and phosphorylation) and their effects on NER (Figure 1).

### Histone Acetylation and NER

Histone acetylation, one of the most important and highly studied histone modifications, is the covalent addition of an acetyl group to the lysine residue within histones. It is mediated via histone acetyltransferases (HATs), which are divided into three families: GNAT (GCN5 related *N*-acetyltransferase), MYST (MOZ, YBF2/SAS3, SAS2, TIP60), and P300/CBP (Hodawadekar and Marmorstein, 2007). Histone deacetylation, on the other hand, is catalyzed by histone deacetylases (HDACs). Based on function and sequence homology to yeast original proteins, the 18 HDAC enzymes in humans are classified into four classes (Seto and Yoshida, 2014). The class I HDACs, homologous to yeast Rpd3 (reduced potassium dependency 3), include HDAC1, HDAC2, HDAC3, and HDAC8. The class II HDACs comprise HDAC4, HDAC5, HDAC6, HDAC7, HDAC9, and HDAC10 and share homology with yeast Hda1 (histone deacetylase 1). SIRT1, SIRT2, SIRT3, SIRT4, SIRT5, SIRT6, and SIRT7 belong to the class III and have homology to yeast Sir2 (silent information regulator 2), an NAD^+^-dependent enzyme. HDAC11 is assigned to the class IV as it is homologous to neither Rpd3 nor Hda1 yeast enzymes. Histone acetylation generally promotes open chromatin and gene activation because the addition of acetyl groups neutralizes the positive charge of histone lysine residues and thus reduces the strong electrostatic histone-DNA interaction, leading to a more open chromatin structure favorable for the transcriptional machinery. Histone acetylation can also be “read” by bromodomain-containing proteins which recruit positive elongation factors (Roth et al., 2001).

Seminal studies in the 1980s showed that histone hyperacetylation induced by sodium butyrate, an inhibitor of histone HDAC, stimulates NER in UV treated human fibroblasts (Smerdon et al., 1982; Ramanathan and Smerdon, 1989) and that a hyperacetylation phase, followed by a hypoacetylation phase, occurred immediately after UV irradiation (Ramanathan and Smerdon, 1986). Yeast Gcn5, a subunit of the SAGA (Spt-Ada-Gcn5 Acetyltransferase) transcriptional coactivator complex, was then identified to be responsible for H3 (K9 and K14) acetylation and global genomic repair at certain loci but not the whole genome (Teng et al., 2002; Yu et al., 2005). It was later found that Rad16, together with Rad7, mediate the UV-dependent H3 (K9 and K14) acetylation by increasing the occupancy of Gcn5 in chromatin after UV treatment (Teng et al., 2008; Yu et al., 2011). Intriguingly, histone variant Htz1 (H2A.Z in humans) promotes H3 acetylation for efficient NER by using the same way as Rad16 (Yu et al., 2013). Like H3 acetylation, histone H4 acetylation also increases in response to UV irradiation and NuA4 (nucleosome acetyltransferase of histone H4) HAT is important for efficient NER in yeast (Irizar et al., 2010; Hodges et al., 2019). Human GCN5, homologous to the yeast Gcn5, was also found to be involved in H3 acetylation and NER in UV-damaged chromatin by physically interacting with the transcription factor E2F1 (Guo et al., 2010; Guo et al., 2011). Another two transcription coactivators in mammalian cells, CBP (CREB-binding protein) and p300, were shown to participate in NER through their HAT activities and physical interactions with repair proteins such as p53, DDB2, CSB and PCNA (Datta et al., 2001; Hasan et al., 2001; Rubbi and Milner, 2003). H3K56 acetylation, catalyzed by P300/CBP in mammals and by Rtt109 (homolog of P300/CBP) in yeast, plays an important role in genome stability (Driscoll et al., 2007; Han et al., 2007). However, it is dispensable for NER and responsible for the restoration of chromatin structure after completion of NER (Battu et al., 2011). Similarly, H3K14 acetylation, on its own, was found to have no effect on the repair of UV damaged DNA in an elegant *in vitro* study. However, it could facilitate UV damage repair in the presence of RSC (Remodeling the Structure of Chromatin), a chromatin remodeler, by enhancing the interaction between RSC and nucleosome (Duan and Smerdon, 2014). Besides acetylating histones, P300/CBP also interacts and acetylates nonhistone NER factor XPG in a PCNA-p21 dependent manner, which makes the 3′ incision on the damaged DNA strand during dual incision (Tillhon et al., 2012). Thus, P300/CBP may promote NER by acetylating both histones and the essential NER factor XPG. Recently, it was reported that GCN5/PCAF (P300/CBP-associated factor) mediated acetylation of RPA1 (replication protein A1) and acetylation of XPF by TIP60 promote NER (He et al., 2017; Zhao et al., 2017; Wang et al., 2020). Like HATs, HDACs can deacetylate both histones and nonhistone NER factors. For example, HDAC3 deacetylates H3K14 after UV irradiation and this deacetylation of H3K14 facilitates the recruitment of XPC during NER (Kakumu et al., 2017; Nishimoto et al., 2020). Interestingly, HDAC4 itself was recently discovered to bind XPC directly for efficient NER (Li et al., 2020). A previous study has shown that XPA, a rate limiting NER factor, is deacetylated by SIRT1 and this deacetylation is required for optimal NER (Fan and Luo, 2010). However, it was later found that only a small fraction of XPA is acetylated and downregulation of SIRT1 has no measurable effect on overall rate of NER. Instead, XPA and hence NER is regulated by circadian clock at the transcriptional level (Kang et al., 2010; Kang et al., 2011).

With the development of NGS-based methods for genome-wide mapping of DNA damage and repair (Sloan et al., 2018; Li and Sancar, 2020), our understanding of DNA damage formation and repair in diverse chromatin environments has increased significantly. With the aid of XR-seq (Hu et al., 2015; Hu et al., 2019), which purely measures ongoing repair by isolating and sequencing the excision products released during NER, a genome-wide comparison of chromatin states, histone modifications, gene expression and UV damage repair kinetics was performed (Adar et al., 2016). It was found that the earliest repair occurs in active and open chromatin regions, while repair in repressed heterochromatin regions is relatively slow. Regions with H3K27 acetylation, which is associated with active promoters and enhancers, have high levels of early repair. Indeed, UV damage repair super hotspots, which are defined as the earliest-repair sites in the genome, are significantly enriched in both frequently interacting regions (FIREs) and super enhancers (Jiang et al., 2021).

### Histone Methylation and NER

Histone methylation, catalyzed by histone methyltransferases (HMTs), is the process of adding one, two, or three methyl groups to lysine and arginine residues in histone proteins. Methyl groups can also be removed from histone residues by histone demethylases. Depending on which residues are methylated and how many methyl groups are added, histone methylation can either increase or decrease gene transcription activity (Greer and Shi, 2012). The most extensively studied methylation of H3K9, H3K27 and H4K20 is associated with repressed transcription, whereas methylation of H3K4, H3K36, and H3K79 is linked to active transcription. Like histone acetylation, methylation events, which can weaken electrostatic attractions between histone and DNA, lead to unwinding of the DNA followed by recruitment of transcriptional machinery, and thus increase transcription activity. Some histone methylations, however, trigger chromatin compaction and inhibit the access of transcriptional machinery to DNA. For example, H3K9 methylation can recruit HP1 (heterochromatin protein 1) and bind to the highly conserved chromodomain of HP1, resulting in chromatin compaction and gene silencing (Jacobs and Khorasanizadeh, 2002). In *Drosophila melanogaster*, a global decrease of H3K9 trimethylation after UV irradiation was discovered in salivary gland cells (Palomera-Sanchez et al., 2010).

In contrast to histone acetylation, which has been known to function in NER for a long time, the involvement of histone methylation in NER was only recently revealed, and one histone methylation, H3K79, was found to function in NER (Bostelman et al., 2007; Chaudhuri et al., 2009; Tatum and Li, 2011; Zhu et al., 2018). In yeast, the H3K79 methylation is catalyzed by Dot1 with its HMT activity. Both Dot1 and H3K79 methylation are required for efficient global genomic repair but not for TCR (Tatum and Li, 2011). It was found that the role of H3K79 methylation in global genomic repair was unlikely achieved through activating cell cycle checkpoints or regulating the expression of NER genes. Rather, it may serve as a docking site for the recruitment of the NER machinery required for global genomic repair (Gsell et al., 2020). It was later found that DOT1L mediated H3K79 methylation is indeed essential for XPC recruitment and efficient NER after UV irradiation in mammalian cells (Zhu et al., 2018). Nevertheless, another study in mouse embryonic fibroblasts suggested that DOT1L is not implicated in global genomic repair, and that it mainly facilitates the reactivation of RNAP II transcription initiation by securing an open chromatin structure (Oksenych et al., 2013). The difference may be explained by the fact that global genomic repair in rodents is significantly slower than that in humans (van der Horst et al., 1997) and that embryonic stem cells primarily eliminate cells containing massively damaged DNA through apoptosis rather than NER (Li et al., 2019). Besides its role in NER, DOT1L is also critical for transcription elongation, DNA damage response, normal development, and formation of heterochromatin (Wysocki et al., 2005; Wood et al., 2018; Ljungman et al., 2019).

H3K4 trimethylation (H3K4me3) and H3K4 monomethylation (H3K4me1) are associated with actively transcribed promoters and active enhancers, respectively. It was found that DDB2, a DNA damage-binding protein, interacts with and recruits the histone methyltransferase ASH1L (Absent, Small or Homeotic discs 1) to UV lesions leading to deposition of H3K4me3. Similar to H3K79 methylation, H3K4me3 facilitates the stable docking of XPC to DNA damage sites enabling the recruitment of downstream NER proteins by XPC (Balbo Pogliano et al., 2017). Interestingly, another study in yeast showed that H4H75E mutation decreases global genomic repair by impairing the recruitment of Rad4 (XPC in humans) to chromatin after UV irradiation (Selvam et al., 2019). As DDB2 preferentially binds to unmethylated nucleosomes, it is likely that H3K4me3 may promote the DDB2-XPC handover at DNA damage sites (Apelt et al., 2021). Like H3K27 acetylation, excision repair in regions marked by H3K4me3 and H3K4me1 occurs much earlier than those regions marked by repressive histone methylations (Adar et al., 2016). H3K4 methylation was also found to promote the repair of DNA double-strand breaks by the non-homologous end joining pathway (Wei et al., 2018). Whether and how H3K79 methylation and H3K4me3 are coordinated during the NER process needs to be elucidated in future studies.

### Histone Ubiquitylation and NER

Ubiquitylation is the addition of ubiquitin, which consists of 76 amino acids and exists in all eukaryotes, to a substrate protein. This process is through a reversible three-step enzymatic reaction requiring ubiquitin-activating enzymes (E1), ubiquitin-conjugating enzymes (E2), and ubiquitin ligases (E3). During the ubiquitylation, one single ubiquitin (monoubiquitylation) or a chain of ubiquitin (polyubiquitylation) can be added to the substrate protein. For polyubiquitylation, the seven lysine residues (K6, K11, K27, K29, K33, K48, K63) and N-terminal methionine (M1) in ubiquitin serve as linkage points of the ubiquitin chain (Komander and Rape, 2012). It is associated with a wide range of cellular processes such as protein degradation, DNA replication, gene transcription and DNA repair (Welchman et al., 2005). Nevertheless, only polyubiquitylation, mostly K48- and K29-linked polyubiquitylation, marks the substrate protein for degradation by the proteasome. Other polyubiquitylations and monoubiquitylations may regulate other cellular processes including NER (Miranda and Sorkin, 2007; Chauhan et al., 2021). Histone ubiquitylation occurs primarily on H2A (K119) and H2B (K20 in humans and K123 in yeast). Both H2A and H2B are mainly monoubiquitylated and involved in crosstalk with other histone modifications such as histone methylations in a variety of cellular processes (Weake and Workman, 2008). H2A ubiquitylation is generally linked to gene silencing, while H2B ubiquitylation plays a role in both repression and activation of transcription. Ubiquitylation of H2A and H2B has been shown to participate in the process of NER. Meanwhile, a subset of NER-related proteins, such as XPC (Rad4 in yeast), DDB2, CSB, UVSSA and RPB1 (the largest subunit of RNAP II), are also ubiquitylated during NER (Gillette et al., 2006; Borsos et al., 2020; Apelt et al., 2021). For example, recent studies revealed that ELOF1 is required for the ubiquitylation of RPB1 K1268, a key signal for the recruitment of downstream repair factors including UVSSA and TFIIH (van der Weegen et al., 2021). However, how ubiquitylation of NER proteins functions in NER will not be discussed in detail in this review.

In yeast, the monoubiquitylation of H2B K123, catalyzed by Rad6/Bre1 complex, is partially required for global genomic repair. The Paf1 complex, a transcription elongation factor containing five subunits, is required for the catalytic activity of Rad6/Bre1 complex (Wood et al., 2003; Tatum et al., 2011; Tatum and Li, 2011). Interestingly, ubiquitylation of H2B K123 is essential for H3K79 methylation, which is catalyzed by Dot1 and required for global genomic repair but not TCR (Tatum and Li, 2011). Thus, it is likely that H2B ubiquitylation promotes global genomic repair indirectly by enabling the recruitment of Dot1 and the subsequent methylation of H3K79. Indeed, it was revealed that H2B ubiquitylation can regulate chromatin dynamics by enhancing nucleosome stability (Chandrasekharan et al., 2009; Chandrasekharan et al., 2010). Ubiquitylation of H2B is also necessary for H3K4 methylation which is mediated by the methyltransferase Set1-COMPASS and promotes NER in a similar way to H3K79 methylation.

UV-DDB, a heterodimeric complex containing DDB1 and DDB2, is part of a big ubiquitin E3 ligase complex that recognizes damaged chromatin and ubiquitylates core histones at damaged sites (Hannah and Zhou, 2009; Sugasawa, 2009). After UV irradiation, H2A (K118 and K119) and H2B are ubiquitylated by this ubiquitin E3 ligase complex (Kapetanaki et al., 2006; Lan et al., 2012). In addition, ubiquitin ligases Ring1B (Ring2) (Wang et al., 2004; Gracheva et al., 2016) and RNF8 (Marteijn et al., 2009) can also catalyze the H2A ubiquitylation. Ring1B was found to interact with UV-DDB-CUL4 and form a stable complex to ubiquitylate H2A at an early step of damage recognition after UV irradiation (Gracheva et al., 2016). Then, ZRF1, a H2A-ubiquitin binding protein, recognizes and remodels the UV-DDB-CUL4-RING1B complex causing the assembly of the canonical UV-DDB-CUL4 complex. XPC is, then, ubiquitylated by UV-DDB-CUL4 (Sugasawa et al., 2005). In this process, ZRF1 works as a switch protein that regulates XPC ubiquitylation through remodeling of the UV-DDB-CUL4-RING1B complex (Gracheva et al., 2016). It was later discovered that NER involves chromatin reorganization and ZRF1, in combination with XPC, facilitates the relocalization of damaged chromatin to the nucleolus for repair (Chitale and Richly, 2017). In the case of RNF8-mediated H2A ubiquitylation, it occurs after the incision step of NER and RNF8 is essential for the recruitment of downstream factors 53BP1 and BRCA1 (Marteijn et al., 2009; Zhu et al., 2009). Interestingly, RNAP II stalling caused by UV-induced DNA damage triggers H2B deubiquitylation at global level through histone deubiquitylases (Ubp8 and Ubp10) and H2B ubiquitylation level restores gradually during NER in yeast and humans (Mao et al., 2014). Without H2B deubiquitylation, TCR is decreased and RNAP II degradation is increased, suggesting deubiquitylation of H2B can facilitate rescue of RNAP II stalled at UV damage sites through TCR in chromatin (Meas and Mao, 2015).

Histones H3 and H4 can also be ubiquitylated by UV-DDB-CUL4 after UV irradiation, thus destabilizing the nucleosomes and facilitating the recruitment of XPC to DNA damage sites (Wang et al., 2006).

### Histone Phosphorylation and NER

Histone phosphorylation is the addition of a phosphate group to histone residues (serine, threonine and tyrosine) by protein kinases and dephosphorylation is the removal of the phosphate group by phosphatases. The most well studied histone phosphorylation is that of the histone H2AX variant (γ-H2AX) on S139 in mammals (Rogakou et al., 1998) or S129 in yeast (Downs et al., 2000). Upon induction of DNA double strand breaks, histone H2AX is rapidly phosphorylated by ATM (ataxia telangiectasia mutated) and DNA-PKcs (Burma et al., 2001; Fernandez-Capetillo et al., 2004). In the case of UV induced γ-H2AX, the phosphorylation is mainly mediated by the kinase ATR (ataxia telangiectasia and Rad3-related) in the presence of DNA single-strand breaks (Ward and Chen, 2001; Hanasoge and Ljungman, 2007; Matsumoto et al., 2007). Histone phosphorylation reduces the positive charge of the histones, leading to a more open chromatin conformation. Unlike histone acetylation and methylation, histone phosphorylation interplays with other histone modifications and serves as a platform for recruiting factors for downstream cascade of events, such as DNA damage checkpoint activation (Fernandez-Capetillo et al., 2002).

It was shown that γ-H2AX has no significant effect on NER in mammals and yeast (Moore et al., 2007; Revet et al., 2011). Instead, NER plays a key role in the induction of γ-H2AX by generating single-stranded DNA during the repair process (Marti et al., 2006; Hanasoge and Ljungman, 2007; Matsumoto et al., 2007). Single-stranded DNA gap intermediates produced during NER can be extended by EXO1 (exonuclease 1) and coated by RPA (replication protein A), the major eukaryotic single stranded DNA-binding protein, which then recruits ATR and other ATR signaling proteins for ATR activation (Giannattasio et al., 2010; Sertic et al., 2011; Kemp, 2019). It is also likely that the single-stranded excision products released during NER are bound by RPA and involved in ATR kinase signaling pathway (Kemp and Sancar, 2012). Moreover, R-loop formation caused by the stalling of elongating RNAP II at a DNA lesion and DNA replication fork stalling induced by UV damage can activate H2AX phosphorylation (Halicka et al., 2005; Marti et al., 2006; Tresini et al., 2015). Interestingly, other phosphorylated histones, such as H3 (S10 and T11) in mammals and H2A (S122 and T126) in yeast, are dephosphorylated after UV irradiation (Sen and De Benedetti, 2006; Moore et al., 2007; Shimada et al., 2008). Phosphorylation on other histones, including H2B (T129) in yeast, H3 (T45) in humans and H3.3 (S31) in mouse embryonic stem cells, have been identified (Lee et al., 2014; Lee et al., 2015; Martire et al., 2019). However, whether and how histone phosphorylation on these residues is involved in NER remain to be investigated.

## ATP-Dependent Chromatin Remodeling and NER

Besides histone modifications, ATP-dependent chromatin remodeling catalyzed by chromatin remodelers is another way to modulate chromatin structure allowing access of NER machinery to damaged DNA during repair (Nag and Smerdon, 2009; Waters et al., 2015). All the ATP-dependent chromatin remodeling complexes have a common ATPase domain and use the energy released from ATP hydrolysis to slide, eject, or restructure nucleosomes during important biological processes including chromosome assembly and segregation, DNA damage and repair, apoptosis, and cell cycle progression (Wang et al., 2007). ATP-dependent chromatin remodelers in eukaryotes are classified into four families: SWI/SNF (switch defective/sucrose nonfermenting), CHD (chromodomain helicase DNA binding), INO80 (inositol requiring 80), and ISWI (imitation switch). Even though all remodelers share a common ATPase domain, their functions are specific because each remodeler has unique protein domains (e.g., bromodomain and helicase) in their ATPase region (Clapier and Cairns, 2009).

The effect of ATP-dependent chromatin remodelers on NER was first studied in two independent *in vitro* studies. In one study, it was discovered that the *Drosophila* ACF (ATP-utilizing chromatin assembly and remodeling factor), which belongs to ISWI family, facilitates NER of (6-4)PP in the linker DNA region, but not in the nucleosome core region (Ura et al., 2001). In the other study, it was found that the yeast SWI/SNF complex enhances NER of AAF-G (acetylaminofluorene-guanine) in nucleosome core particle (Hara and Sancar, 2002). The different effects might be due to the specific functions for the two families of chromatin remodelers. ISWI remodelers maintain high order of chromatin structure by creating equal spacing between nucleosomes, while SWI/SNF remodelers rearrange nucleosomes through unwrapping, sliding, or ejecting nucleosomes (Clapier and Cairns, 2009). Similarly, the human CSB (cockayne syndrome protein B), which belongs to SWI/SNF family and is essential for TCR, was found to remodel chromatin *in vitro* (Citterio et al., 2000).

Like *in vitro* studies, one subsequent *in vivo* study in yeast showed that the SWI/SNF chromatin remodeling complex interacts with Rad4-Rad23 heterodimer to increase DNA accessibility for NER upon UV irradiation (Gong et al., 2006). In humans, SWI/SNF complex was also found to associate with XPC at DNA damage sites and promote recruitment of ATM and NER factors (e.g., XPG) in response to UV irradiation (Ray et al., 2009; Zhao et al., 2009). Rad16, another SWI/SNF chromatin remodeler, was found to promote efficient global genomic repair by increasing the occupancy of Gcn5 in chromatin and subsequent H3 acetylation through its ATPase and RING domains (Yu et al., 2011). The INO80 family of chromatin remodelers modulate chromatin structure in different ways including exchange of histone variants (e.g., H2AZ) and nucleosome sliding (Clapier et al., 2017). By using the energy from ATP hydrolysis, it can incorporate and remove histone variants in the nucleosome and create nucleosome-free regions. In humans, it was found that INO80 is recruited to UV induced DNA damage sites independent of XPC and interacts with DDB1, suggesting a role in the initiating step of NER (Jiang et al., 2010). One study, however, reported that yeast INO80 is recruited to chromatin by Rad4 upon UV irradiation and restores chromatin structure after NER (Sarkar et al., 2010). Another study in yeast showed that cells without INO80 are proficient in repair of CPDs and replication defects may contribute to UV sensitivity observed in cells lacking INO80 (Czaja et al., 2010). CHD family remodelers are primarily responsible for transcriptional repression by assembling nucleosomes, although certain CHDs in higher organisms can slide or eject nucleosomes to promote transcription (Clapier et al., 2017). It was found that human CHD1 is recruited to UV damage sites in an XPC dependent manner and mediates XPC-to-TFIIH handover to facilitate NER in chromatin (Ruthemann et al., 2017).

Recent development of NGS-based sequencing methods has enabled researchers to capture the spatial genome organization and map DNA damage formation and repair across the whole genome (Garcia-Nieto et al., 2017; Sloan et al., 2018; Li and Sancar, 2020; Sanders et al., 2020). Studies in yeast showed that the global genomic repair complex (Rad16-Rad7-Abf1), which includes SWI/SNF chromatin remodeler Rad16, binds to the boundary sites of chromosomally interacting domains (CIDs) frequently and regulates distribution of histone H3 acetylation upon UV irradiation. The global genomic repair complexes initiate nucleosome remodeling in the vicinity of their binding sites in response to UV damage, which defines the origins of NER in chromatin (Yu et al., 2016; van Eijk et al., 2019). A recent study reported that SWI/SNF is not generally required for efficient NER and only affects NER at certain genes in yeast, while RSC (chromatin structure remodeling), another SWI/SNF family chromatin remodeler, is required for NER throughout the yeast whole genome (Bohm et al., 2021).

## Conclusion

NER is a highly conserved and versatile DNA damage removal pathway that counteracts challenges from a variety of DNA damaging agents. Since the basic molecular mechanism of NER has been well studied by using *in vitro* experimental system (Sancar, 2016), it becomes more and more intriguing to explore and decipher the mysteries of the NER process within the complex and dynamic molecular environment of the cell. This review is focused on our current understanding of epigenetic regulation of NER, especially on how different histone modifications affect the *in vivo* NER process in the context of chromatin. Upon DNA damage induction, a cascade of cellular events, including DNA damage checkpoint activation, histone modifications, chromatin reorganization, DNA repair, and apoptosis, would occur to deal with the assault.

In recent years, we have gained relatively better understanding of how DNA damage is recognized by NER related machinery such as UV-DDB and XPC-RAD23B-CETN2 in the chromatin environment (Apelt et al., 2021). However, how different epigenetic factors, including chromatin modifications, nucleosome positioning, chromatin remodeling, DNA modifications and non-coding RNA, are mechanistically orchestrated to give NER machinery access to DNA lesions is still unknown. These factors may interplay with each other, complicating the delicate NER process. Even for modification of histones itself, one modification may promote or repress other modification(s). The modifications on histones and non-histone proteins may indirectly regulate NER through altering gene expression profile, which increases the complexity of studying NER. Another open question is how the cellular chromatin organization is restored after the NER process. The development and application of novel research methods such as cryo-EM and third-generation sequencing would aid in our in-depth understanding of these questions.
